# Pazopanib as first line treatment for solitary fibrous tumours: the Royal Marsden Hospital experience

**DOI:** 10.1186/s13569-015-0022-2

**Published:** 2015-02-02

**Authors:** Marco Maruzzo, Juan Martin-Liberal, Christina Messiou, Aisha Miah, Khin Thway, Rolyn Alvarado, Ian Judson, Charlotte Benson

**Affiliations:** Sarcoma Unit, The Royal Marsden NHS Foundation Trust, Fulham Road, SW3 6JJ London, UK; Radiology Department, The Royal Marsden NHS Foundation Trust, London, UK; Pathology Department, The Royal Marsden NHS Foundation Trust, London, UK

**Keywords:** Pazopanib, Solitary fibrous tumour, SFT, Sarcoma

## Abstract

**Background:**

Solitary Fibrous Tumour (SFT) is a rare soft tissue neoplasm, described in several locations in the body. It is classified as intermediate malignant potential with low risk of metastasis and has a low tendency to recur after primary surgery.

**Methods:**

We performed a prospective data collection of the patients with SFT presented to the Royal Marsden Hospital from January to December 2013, and treated with pazopanib in first line. Demographics, anatomic primary sites, treatment and survival outcomes were collected from patients’ electronic records.

**Results:**

13 patients (54% females) were identified with a median age of 51 years (range 37–77). Most of the patients (77%) were diagnosed with extra-thoracic SFT. All the patients received first line treatment with pazopanib for metastatic disease. Median overall survival (OS) was 13.3 months. Median progression free survival (PFS) was 4.7 months. No statistically significant difference was found in OS and PFS between primary thoracic SFT and primary extra-thoracic SFT. According to RECIST, one partial response (9%) and eight disease stabilizations (73%) were found as best responses. Using Choi criteria, there were 5 partial responses (46%) and 4 stabilizations (36%).

**Conclusion:**

Our prospective data confirm that anti-angiogenic drugs are active in SFT. PFS and overall response do not appear significantly lower than other reported series on the same disease. Furthermore, pazopanib is a drug already licensed in soft tissue sarcomas and these data suggest its activity also in this particular subtype of sarcomas.

## Introduction

Solitary Fibrous Tumour (SFT) is a rare soft tissue neoplasm, initially thought to occur exclusively within the thorax [1] and now known to arise from all anatomical sites [2]. In the past, SFT has also been called hemangiopericytoma, a term used over the years to describe a wide variety of tumours with some common morphological characteristics. Different biological entities have progressively been identified for this category, and most of them are now recognized as SFTs [3].

Recently, STFs have been described in several locations also outside the thoracic cavity, including head and neck, abdomen, retroperitoneum, and other soft tissue sites [4-6].

SFTs are classified as having intermediate malignant potential with low risk of metastasis under the WHO classification [7] and they have a low tendency to recur after primary surgery [8]. However, the clinical behaviour is hard to predict and several prognostic factors have been considered in order to assess the behaviour of the disease. In a recent analysis of a large cohort of SFTs, the size and the mitotic index have been proposed as factors to consider after primary surgery which may help to stratify the follow-up of the patients that might have an increased risk of recurrence [5]. Generally, treatment for metastatic SFTs is not curative and is of palliative intent.

The role of chemotherapy has been explored in several small series, with conflicting results but overall indicating limited efficacy [9-12]. Recently, the role of dacarbazine in SFT has been investigated within a large mono-institutional case series with positive results [13]. Also the activity of temozolamide and bevacizuamb has been reported [14] and other antiangiogenic drugs have shown some activity, such as sorafenib [15] and sunitinib [16].

Pazopanib is as a second-generation small-molecule, potent and selective multi-targeted receptor tyrosine kinase inhibitor (TKI) active against vascular endothelial growth factor receptors (VEGFR) 1, 2, and 3, platelet-derived growth factor receptors (PFGFR), and KIT. It also has modest activity against fibroblast growth factor receptors (FGFR) 1, 2, and 3 [17]. Its activity blocks tumour growth and inhibits angiogenesis. It is now approved for first line treatment in kidney cancer [18] and for second or further line in non-adipocytic soft tissue sarcomas after failure of previous chemotherapy [19].

In this paper we report our experience on the management of SFT with a group of 13 patients prospectively collected and treated with pazopanib as a first line treatment for metastatic disease.

## Methods

We prospectively collected data from all consecutive patients with histologically confirmed advanced SFT, who were treated with pazopanib as first-line systemic treatment at the Royal Marsden Hospital, from January to December 2013. Demographics, site, treatment, toxicities and survival outcomes were collected from electronic patients’ records. Toxicity was recorded according to CTCAE v4.0 criteria, while disease response was assessed with repeated computerised tomography (CT) scan and or magnetic resonance imaging (MRI) every two or three months. Response was assessed according to RECIST version 1.1 [20] and Choi criteria [21] by a named sarcoma radiologist (CM). Histological diagnosis of SFT was reviewed and confirmed in all cases by an experienced sarcoma pathologist.

Ethical approval was provided by relevant Committee at the Royal Marsden Hospital.

For statistical analysis, progression free survival (PFS) was defined from date of starting chemotherapy to date of progression or death where any progression free surviving patients were censored at last follow up; overall survival (OS) was defined from date of starting chemotherapy to date of death where any surviving patients were censored at last follow up. Statistical analysis was conducted with Kaplan-Meier method by a designated Sarcoma Unit statistician.

## Results

### Demographics

Thirteen consecutive patients were seen at the Royal Marsden Hospital in 2013 with diagnosis of symptomatic metastatic SFT and treated with pazopanib. The majority of them were women (54%) with a median age of 51 years (range 37–77). Most of the patients (77%) were diagnosed with extra-thoracic SFT; predominant primary sites were abdomen, pelvis, spine, thighs and thorax. All the patients had an ECOG (Eastern Cooperative Oncology Group) performance status between 0 and 2. All the patients had documented radiological progressive disease within three months prior to starting on pazopanib. Patient demographics and prior management are summarised in Table 1.

**Table 1:** **Patients’ characteristics and prior management**

**Age** median (range)	51 (32–77)
**Sex**	
Male	6 (46%)
Female	7 (54%)
**ECOG PS**	
0	2 (15%)
1	9 (70%)
2	2 (15%)
**Primary location**	
Thorax	4 (31%)
Abdomen	3 (24%)
Pelvis	2 (15%)
Thigh	2 (15%)
Others	2 (15%)
**Prior surgery**	
Yes	8 (62%)
No	5 (38%)
**Metastatic disease**	
Yes	10 (77%)
No	3 (23%)
**Site of metastases**	
Lungs	4 (31%)
Liver	3 (24%)
Bone	2 (15%)
Intra-abdominal	2 (15%)
Kidney	1 (8%)
**Disease progression**	
before pazopanib	13 (100%)

### Treatment

Eight patients (72%) underwent surgery of the primary tumour; one of them received several surgical resections of primary disease and small single metastases. Five patients (28%) were deemed unresectable at presentation. All the patients received first line pazopanib for metastatic disease. 8 (62%) patients started with full dose of pazopanib (800 mg daily), 5 (38%) with a reduced dose of 600 mg daily based on physicians’ assessment, mainly because of performance status. Three patients (23%) required dose reduction because of grade 3 or persistent grade 2 side effects, no-one required more than one dose reduction during the treatment. The median duration of the treatment was 4.1 months (range 0.95-22.2). 85% of the patients (11) were discontinued from the treatment because of disease progression (9, 69%) or toxicities (2, 15%). Two patients (15%) are still on the treatment at the time of this report. Treatment response is summarized in Table 2.

**Table 2:** **Best response to treatment**

**No.**	**Gender**	**Age**	**Best Response per RECIST**	**Best Response per CHOI**	**Tx duration (weeks)**
1	M	45	not assessable	not assessable	4.1
2	M	46	SD	SD	39.3
3	F	73	SD	PR	10.7
4	F	69	PD	PD	13.0
5	F	57	not assessable	not assessable	2.0
6	M	49	PD	PD	7.0
7	F	68	PR	PR	20.3
8	M	37	SD	SD	95.1
9	F	66	SD	PR	60.0
10	F	37	SD	PR	19.7
11	M	32	SD	PR	17.4
12	M	77	SD	SD	10.0
13	F	51	SD	SD	21.9

### Toxicities

All the patients experienced grade 1 or 2 toxicities. Grade 3 to 4 toxicities were reported sporadically: one patient (7%) experienced haemoperitoneum and another two (15%) had intratumoural bleeding, one patient (7%) had grade 3 liver function test alteration, which resolved with dose reduction, one experienced a grade 3 hand foot skin reaction, one patient (7%) had multiple kidney infarcts without renal function alteration. Two patients discontinued the treatment because of severe adverse events: one because of massive haemoperitoneum, the second one because of grade 3 skin toxicity. No toxic deaths were reported. All the toxicities are summarized in Table 3.

**Table 3:** **Treatment related toxicities (CTCAE version 4.0)**

	**G1/G2**	**G3/G4**
Intratumoral bleeding	-	1 *(7%)*
Haemoperitoneum	-	1 *(7%)*
Multiple kidney infarct	-	1 *(7%)*
Skin reactions	5 *(38%)*	1 *(7%)*
Fatigue	10 *(77%)*	-
Diarrhoea	5 *(38%)*	-
Dysgeusia	4 *(31%)*	-
Nausea	3 *(24%)*	-
Mucositis	3 *(24%)*	-
Anorexia	3 *(24%)*	-
Liver function test alteration	*2 (15%)*	-
Neutropenia	1 *(7%)*	-

### Radiological response

Eleven out of 13 patients were assessable for response according to RECIST and Choi criteria. According to RECIST, the assessments showed one partial response (9%), eight disease stabilizations (73%), and two disease progression (18%) as best responses. Compared to these results, with Choi criteria there was a greater number of partial responses (5, 46%), a smaller number of stable diseases (4, 36%), and the same number of progressions (2, 18%). Two patients were not assessable either with RECIST or Choi criteria because of early discontinuation before radiological assessment due to severe side effects.

### Survival

**Figure 1:**
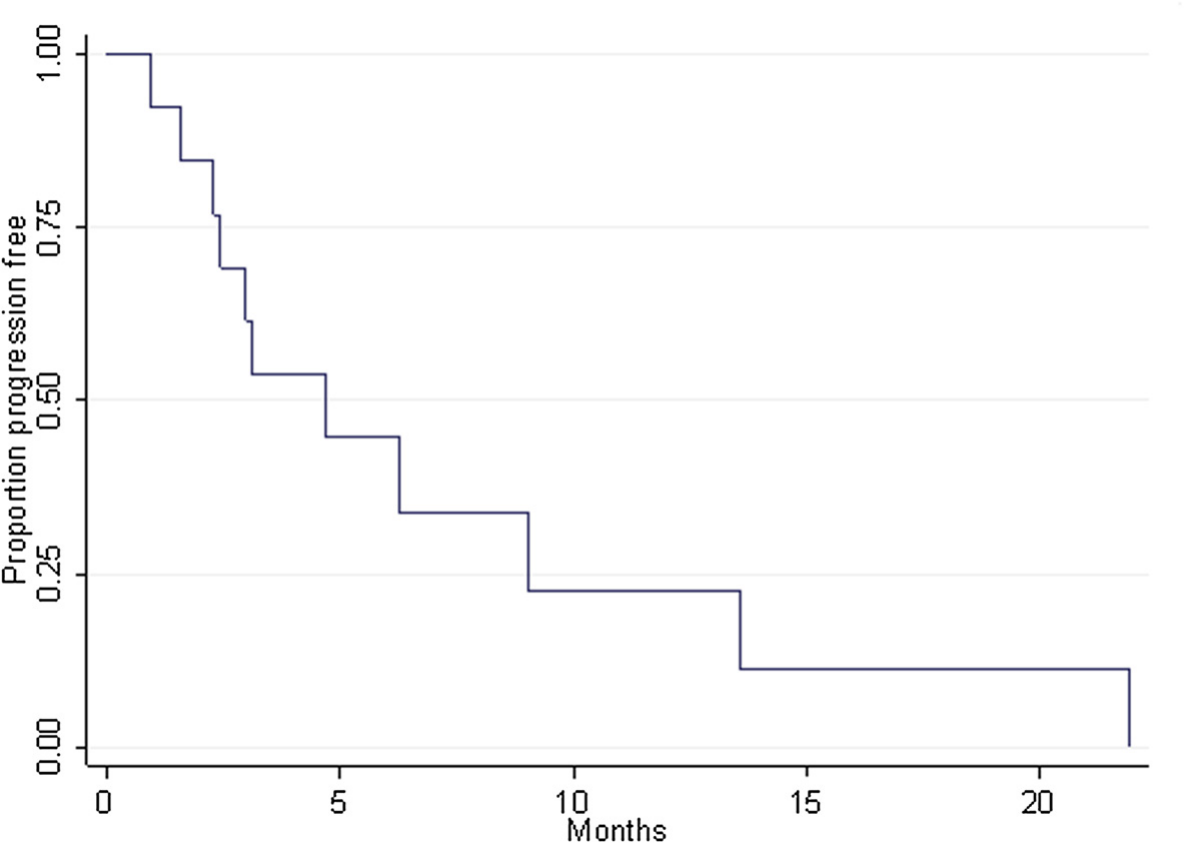
**Progression free survival: median rate 4.7 months (95% CI 1.8 – 7.4); 6-month rate 44.9% (95% CI 17.7 – 69.0).**

**Figure 2:**
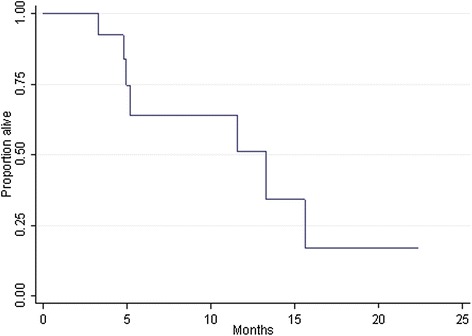
**Overall Survival median rate 13.3 months (95% CI 3.9 – 22.6); 6-month rate 63.9% (95% CI 29.3 – 85.0).**

11 out of 13 patients progressed during the treatment with pazopanib and were discontinued. For two patients the treatment is still ongoing. With a median follow-up time of 12.3 months, median progression free survival (PFS) was 4.7 months (95% CI 4.8-7.4). The 6-month progression-free rate was 44.9% (Figure 1). Median overall survival (OS) was 13.3 months (95% CI 3.9-22.6). The 6-month overall surival rate was 66.9% (Figure 2).

Median PFS and OS for the subgroup of primary thoracic SFT were 9.0 and 11.5 months respectively, compared to 3.1 and 13.3 months respectively in the primary extra-thoracic subgroup, with no statistical significance in the comparison (p = 0.773 and p = 0.437, respectively).

## Discussion

Since January 2013, 13 patients with progressive SFT have been treated at the Royal Marsden Hospital with continuous dosing pazopanib. This is a prospective collection of data of all consecutive patients treated in the same way in a single institution. Among the 11 patients assessable for response, the clinical benefit rate (partial response + disease stabilisation) was the same (82%) using both RECIST and Choi criteria. The outcome of the patients in our series suggests that pazopanib is an active treatment in advanced SFT. This is consistent with the results achieved with other anti-angiogenic therapies already reported in the literature. However, our series show some significative differences.

For instance, Stacchiotti et al. [16] focused on the treatment of SFT with sunitinib. Among a large cohort of 31 patients, they found a larger number with progressive disease compared to this series, and a lower overall response rate. However, interestingly, in that series the median PFS was 6.0 months whereas it was 4.7 months in our patients. Similarly to us, they found sunitinib to be active in SFT, with possible long lasting disease control.

The French Sarcoma Group recently reported their experience with sorafenib, another small molecule antiangiogenic drug [22]. Among five patients, they found no objective response, with a median overall survival of less than 20 months, but with some evidence of disease stabilisation.

Similar results were reported from a single cancer centre, 10 patients received sunitinib or pazopanib as a second or further line of treatment, with a median PFS of 5.2 months and overall an acceptable toxicity profile [23].

The MD Anderson Cancer Centre reported on the efficacy of an anti-angiogenetic drug combination for SFT [14]. Temozolamide plus bevacizumab produced a Choi partial response in 11 patients (79%) with hemangiopericytoma and malignant SFT, two patients (14%) had stable disease and one patient (7%) had progressive disease. The estimated median PFS was 9.7 months with a 6-month progression-free rate of 78.6%.

The role of chemotherapy has also been investigated. Our group [12] reported of 17 SFT patients treated with anthracycline-based chemotherapy, in whom there was only 1 partial response, and most of the patients had progressive disease. The median PFS was 4.2 months and OS was 14.6 months. The study was interpreted as showing that chemotherapy was of little value in advanced SFT, however the median PFS, OS and likelihood of response according to RECIST were little different from the results with pazopanib, emphasising the need for caution in interpreting the results from small series, and demonstrating the need for larger, prospective phase II trials in this disease.

Another group of authors has reported on the outcome of SFT treated with dacarbazine [13] showing the antitumor activity of this drug in SFT. Among 8 patients treated, they found 3 partial responses and only one progressive disease, with a median PFS of 7 months.

The same French group who reported on antiangiogenic drugs, also reported their experience with chemotherapy [23]. They treated 23 patients in first line with different chemotherapy regimens, mainly containing anthracycline (18 out of 23). The median PFS was 5.1 months, with 2 partial responses (9%) and 13 stabilizations (57%) as best response.

A comparison among the survival outcome from all the studies conducted so far for metastatic SFT is reported in Table 4.

**Table 4:** **Published experience of systemic therapy in metastatic/locally advance SFT**

	**Pts**	**RR with RECIST**	**RR with Choi**	**OS (mos)**	**PFS (mos)**
Sunitinib (*Stacchiotti*)	31	6.4%	NR	16.0	6.0
Sorafenib (*FSG*)	5	0%	NR	19.7	NR
Antiangiogenic drugs (*Levard*)	10	0%	NR	NR	5.1
Temozolamide and Bevacizumab (*Park*)	14	NR	79%	NR	9.7
Dacarbazine (*Stacchiotti*)	8	37.5%	NR	NR	7.0
Anthracycline (*Costantinidou)*	17	5.8%	NR	14.6	4.2
Chemotherapy (*Levard*)	23	9%	NR	NR	5.2
Pazopanib (*present study*)	13	9%	46%	13.3	4.7

(mos = months, NR = not reported, pts = number of patients, RR = response rate).

**Figure 3:**
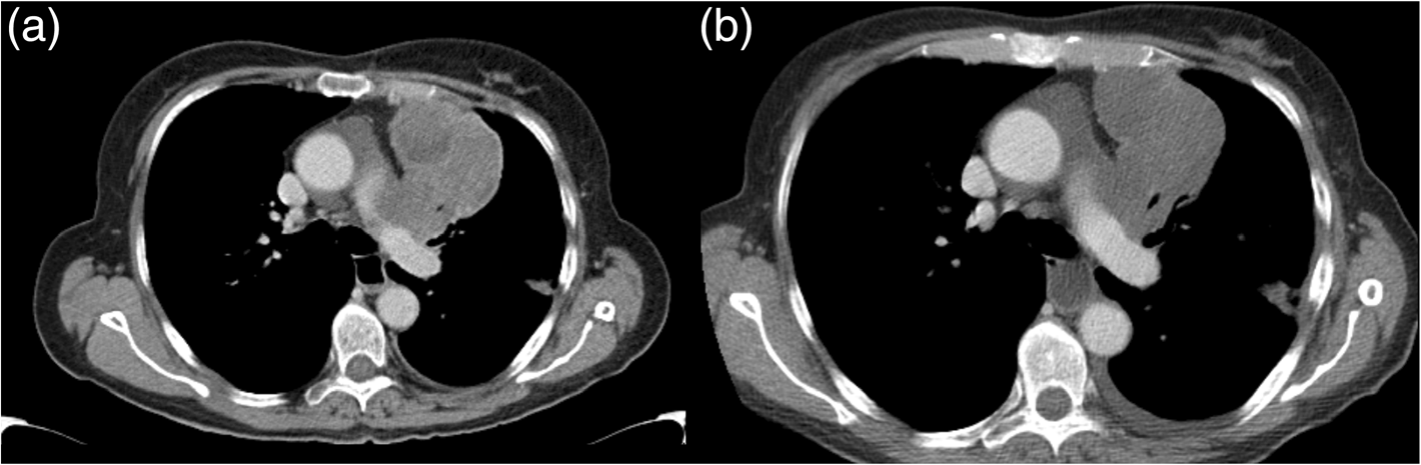
**An example of Choi response to the treatment: (a) baseline HU 43; (b) after six months of treatment HU 35.**

In summary, and compared to the largest recent series reported, our data confirm that pazopanib has some activity against SFT. A number of agents appear to be capable of inducing genuine disease stabilisation. Our experience and that of the group at MD Anderson suggests that consideration should be given to evaluation of response using Choi criteria rather than RECIST. Several studies suggested that Choi could be a more sensitive tool to evaluate disease response in sarcoma, especially in gastrointestinal stromal tumours [21,24,25], and there is some evidence for its use in non GIST sarcomas [26,27]. A significant obstacle to the widespread introduction of these criteria is the limited reproducibility. However some of the challenges of Choi criteria such as the need to avoid areas of fistula and obtaining reproducible measurements on mobile small bowel lesions are more common in GIST and use in non GIST sarcomas is therefore often less problematic. The use of Choi criteria, with the double assessment of density and size, requires increased time of an experienced sarcoma radiologist to report all examinations for the same patient and consistent CT protocols at each follow-up time point. Implementation of Choi criteria in a non-oncologic hospital, with lack of expertise may not be possible. Anyway, Choi criteria were overall able to better appreciate the response, since they are based on changes in contrast enhancement on CT, which reflects tumour blood flow, as well as tumour size, which may not alter greatly with these agents. We assessed the activity of pazopanib both with RECIST and Choi. Since Choi criteria are more stringent in the definition of progression, we were not concerned about failing to identify treatment failure. and indeed when RECIST showed progressive disease this was confirmed by Choi. Similarly, in 4 cases (36%) of stable disease by RECIST, partial response could be detected by Choi, with a significant reduction of attenuation on CT (Figure 3). For future studies Choi criteria should be incorporated as part of the disease assessment as well as RECIST as a better indication of disease modulation by agents such as pazopanib.

In terms of toxicity, pazopanib appeared to be an acceptable treatment. Most of our patients experienced grade 1 to 2 toxicities and only a few of them had severe side effects The toxicity profile in our cohort of SFT is in line with the result from the phase III registration trial of pazopanib in soft tissue sarcoma [19]. The most common adverse events reported with pazopanib in treating sarcomas were fatigue, diarrhoea, nausea, weight loss, and hypertension. From our series, fatigue, diarrhoea, and skin reaction were the most common side effects. Interestingly, only a small number of grade 3 or 4 side effects were observed.

In terms of further research, a phase II trial, to be performed by the Spanish Sarcoma Group together with French and Italian Centres, is due to investigate the role of pazopanib in SFT (NCT 02066285). An American study in advanced sarcoma with dasatinib included patients with SFT (NCT 00464620) and final data are awaited. We are recruiting to a translational research study with pazopanib which will include patients with SFT utilizing tumour biopsies. In this way, we want to search for molecular targets or specific pathways able to help in understanding which patients could most benefit from these treatments.

Overall, our data confirm that anti-angiogenic agents have some activity against SFT. Pazopanib is licensed for the treatment of soft tissue sarcomas and hence is available for treating this particular subtype. However, in spite of the evidence that disease stabilisation and sometimes objective response can be achieved in a percentage of patients, median PFS and OS are remarkably similar across the various reports indicating the need for a more effective approach to the systemic management of this disease.
